# Skin optical properties in the obese and their relation to body mass index: a review

**DOI:** 10.1117/1.JBO.27.3.030902

**Published:** 2022-03-29

**Authors:** Andres J. Rodriguez, Mel Tananant Boonya-Ananta, Mariacarla Gonzalez, Vinh Nguyen Du Le, Jesse Fine, Cristina Palacios, Mike J. McShane, Gerard L. Coté, Jessica C. Ramella-Roman

**Affiliations:** a Florida International University, Department of Biomedical Engineering, Miami, Florida, United States; b Texas A&M University, Department of Biomedical Engineering, College Station, Texas, United States; c Florida International University, Robert Stempel College of Public Health and Social Work, Miami, Florida, United States; d Texas A&M University, TEES Center for Remote Health Technologies and Systems, College Station, Texas, United States; e Texas A&M University, Department of Material Science and Engineering, College Station, Texas, United States; f Florida International University, Herbert Wertheim College of Medicine, Miami, Florida, United States

**Keywords:** optical properties, obesity, skin, body mass index, biophotonics, wearables

## Abstract

**Significance:**

Obesity is a worldwide epidemic contributing directly to several cardiovascular risk factors including hypertension and type 2 diabetes. Wearable devices are becoming better at quantifying biomarkers relevant for the management of health and fitness. Unfortunately, both anecdotal evidence and recent studies indicate that some wearables have higher levels of error when utilized by populations with darker skin tones and high body mass index (BMI). There is an urgent need for a better evaluation of the limits of wearable health technologies when used by obese individuals.

**Aims:**

(1) To review the current know-how on changes due to obesity in the skin epidermis, dermis, and subcutis that could affect the skin optical properties; (2) for the green wavelength range, to evaluate the difference in absorption and scattering coefficients from the abdominal skin between individuals with and without elevated BMI. The changes include alterations in layer thickness and cell size, as well as significant differences in chromophores and scatterer content, e.g., water, hemoglobin, collagen, and lipids.

**Approach:**

We have summarized literature pertaining to changes in skin and its components in obesity and report the results of our search using articles published between years 1971 and 2020. A linear model was used to demonstrate the absorption and reduced scattering coefficient of the abdominal skin of individuals with and without elevated BMI in the green wavelength range (530 to 550 nm) that is typically found in most wearables.

**Results:**

The general trends indicate a decrease in absorption for both dermis and subcutis and an increase in reduced scattering for both epidermis and dermis. At 544-nm wavelength, a typical wavelength used for photoplethysmography (PPG), the absorption coefficient’s relative percentage difference between high and low BMI skin, was 49% in the subcutis, 19% in the dermis, and negligible in the epidermis, whereas the reduced scattering coefficient relative difference was 21%, 29%, and 165% respectively.

**Conclusions:**

These findings suggest that there could be significant errors in the output of optical devices used for monitoring health and fitness if changes due to obesity are not accounted for in their design.

## 1 Introduction

### 1.1 Background

Obesity is a worldwide epidemic, which has increased over the past 45 years by >7%, 8%, and 9% in children, men, and women, respectively.^1^ In the U.S. alone, obesity prevalence has risen consistently and half of the adult and adolescent population is expected to be either obese or overweight in 10 years.^2^ Obesity trends vary across regions, ethnicities, and socioeconomic status.^3^ In particular, individuals of darker skin (Blacks and many Hispanics) have a higher prevalence of obesity compared with whites, which has been increasing steadily over the years.^4^ Both the Centers for Disease Control and Prevention and the National Institutes of Health categorized obesity as a comorbidity and risk factor to many other diseases including type 2 diabetes, cardiovascular disease, and high blood pressure, among others.^5^ Thus, obesity has been associated with higher healthcare costs in both the government and private sector.^5^^,^^6^

Modern attempts at reducing the prevalence of obesity and its comorbidities include the use of mobile applications and wearable devices by promoting physical activity and counting caloric intake.^7^^–^^9^ Many of these technologies rely on tracking activity and other biomarkers, such as heart rate and blood oxygenation via body-worn sensors, many of which are optical in nature. However, developing optical devices for use on obese populations requires an understanding of the anatomical and physiological changes that occur as a person moves from low to high body mass index (BMI) levels and how those changes affect the optical interactions. Hence, an in-depth study of light interaction with tissues of the obese is crucial in developing optical devices for this population.

In this paper, we perform a comprehensive review of skin anatomy and its layered-specific anatomical changes and physiological changes linked to obesity and how these changes affect the optical properties of each layer. We then introduce a model to extrapolate the optical properties in relation to the changes in BMI.

### 1.2 Basic Skin Anatomy Spectral Characteristics

The human skin can be divided into three layers: the stratified cellular epidermis, the dermis of connective tissues, and the subcutis.^10^^,^^11^ The epidermis can be further divided into five layers: stratum corneum, granular layer, spinous layer, basal layer, and basement membrane.^10^^–^^12^

**Fig. 1:**
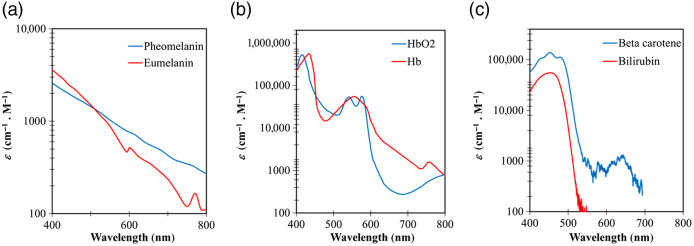
Extinction coefficient spectrum for major absorbers present in human skin: (a) pheomelanin and eumelanin, (b) oxygenated hemoglobin (${\mathrm{HbO}}_{2}$) and deoxygenated hemoglobin (Hb), and (c) beta carotene and bilirubin from Ref. 22.

As shown in Table 1, the total thickness of the epidermis ranges from 0.01 to 0.15 mm, depending on age, gender, lifestyles, regions within the body, and health status.^13^^–^^17^ The stratum corneum is ∼0.01- to 0.02-mm thick^18^ and is made of keratinocytes embedded in a lipid matrix.^19^ Along with keratinocytes, other cell types found in the other epidermal sublayers are melanocytes. There are two types of melanin that act as absorbers: phaeomelanin and eumelanin; the latter associates with skin color.^20^^,^^21^ The absorption spectrum of phaeomelanin and eumelanin is broad [Fig. 1(a)], and the ratio between their concentrations ranges between 0.049 and 0.36.^23^ In general, melanin determines the absorption property of the epidermis, and melanin absorption level depends on the volume fraction of melanosomes in the epidermis, which typically ranges from 1.3% to 43%^24^ as depicted in Table 1. The absorbance of melanin has been well documented, showing the strongest absorption in the UV-short visible (VIS) range.^18^^,^^25^^–^^30^ Beyond the absorbance from the two types of melanin, water and lipid also factor into the absorbance in the epidermal layers.

**Table 1:** Summary of skin components.^13^^–^^17^

	Epidermis	Dermis	Subcutis
Thickness (mm)	0.01 to 0.15	1 to 3	1.65 to 18.2
Cell types	Keratinocytes	Fibroblasts	Adipocytes
	Melanocytes	Mast cells	Fibroblasts
	Langerhans	Stem cells	
	Merkel cells		
Absorbers	Phaeomelanin	Hemoglobin	Hemoglobin
	Eumelanin	Bilirubin	Lipids (fat)
	Water	Water	Water
	Lipid	Beta-carotene	
Scatterers	Cells/nuclei/organelles	Collagen	Collagen
			Lipids (fat)
Fluorophores	Keratin, tryptophan, melanin retinol	Tryptophan, tyrosine, flavins, lipofuscin, vitamin A, NADPH, NADP, FAD, collagen, elastin, AGEs, vitamin B, hemoglobin	Collagen, elastin, AGEs, vitamin A, hemoglobin

The dermis thickness ranges from 1 to 3 mm^13^^,^^14^ (Table 1) and is primarily composed of dense, irregular connective tissue with nerves and blood vessels (capillaries). The cell types in this layer are typically fibroblasts, mast cells, and stem cells. The volume fraction of blood in the dermis ranges from 0.2% to 7%.^24^ Hemoglobin concentration in whole blood is between 120 and 130  g/L.^31^ Blood in the arteries is rich with oxygenated hemoglobin (${\mathrm{SaO}}_{2}$ 90% to 100%), whereas venous blood generally has lower oxygenated hemoglobin (${\mathrm{SvO}}_{2}>50\%$).^32^^,^^33^ The absorption spectrum of oxygenated and deoxygenated hemoglobin is shown in Fig. 1(b). In addition to hemoglobin, bilirubin and beta-carotene are the other two primary absorbers found in the blood in the dermal capillaries [Fig. 1(c)]. The latter two contribute to the yellow/orange tint of human skin and their spectral similarity may pose an issue to optical instruments aimed at measuring their respective concentrations. The dermis extracellular matrix is mainly composed of collagen, a protein made by fibroblasts.^34^ Collagen fibrils are strong scatterers.

The subcutis, the innermost layer of the skin, is also known as the hypodermis or subcutaneous layer and consists of a network of fat cells (adipocytes), fibroblasts, and collagen. The subcutis functions as the body energy storage, heat insulator, and shock-absorber. Blood vessels, nerves, lymph vessels, and hair follicles are found in the subcutis. The thickness of the subcutis layer varies throughout the body and from person to person, ranging from 1.65 to 14.65 mm in males to 3.3 to 18.2 mm in females, and increases as the BMI increases.^35^ Absorption within the human subcutaneous layer is defined primarily by hemoglobin, fat (lipids), and water.^36^^,^^37^

Select tissue constituents can emit fluorescent light after encountering incident excitation of light of a shorter wavelength. The emitted light from these endogenous fluorophores combines and leads to overall tissue autofluorescence. The observed emission spectrum is largely a function of the excitation wavelength and spectral analyses can be used to quantify the presence or absence of endogenous fluorophores.^38^^–^^40^ Within the visible wavelength excitation range, the most common fluorophores are collagen, elastin, nicotinamide adenine dinucleotide phosphate (NADPH, bound, and free), Flavin adenine dinucleotide (FAD), vitamin A (retinol), flavins, porphyrins, keratin, lipofuscin, melanin, tryptophan, and tyrosine^41^^–^^44^

## 2 Differences in the Primary Layers of the Skin with Obesity

As depicted in the Supplemental Materials and articulated below, there are several factors within the three primary layers of skin that change due to obesity.

### 2.1 Epidermis

#### 2.1.1 Melanin

Different types of melanin react differently to UV radiation. For example, eumelanin behaves as a photoprotector, where pheomelanin can generate reactive oxygen species (ROS).^45^ Randhawa et al.,^46^ showed the presence of eumelanin pigments in the periphery of adipocytes of severely obese patients, for nonobese individuals they showed few or none.

#### 2.1.2 Thickness

Epidermal thickness has been shown to increase with increasing BMI.^47^^,^^48^ Altintas et al.^47^ revealed through histology that there was 20% thicker epidermis in the forearm of overweight individuals versus their control group (54.8 versus 44  μm). Epidermal skin appears thicker and rougher in the obese. Among American women, skin roughness is strongly correlated to BMI, as it increases by 70% among the skin of the obese versus the nonobese.^49^ Another study by Horie et al. looked at breast tissue in Japanese women categorized as a control group or obese group, defined as ($\mathrm{BMI}<25\;\mathrm{kg}/m^{2}$) or ($25\;\mathrm{kg}/m^{2}\leq \mathrm{BMI}<35\;\mathrm{kg}/m^{2}$), respectively. In the obese group, structural changes include epidermal thickening and keratinocyte proliferation. Both skin cholesterol and fatty acid levels exhibited an “inverted-U” relationship with BMI, suggesting that there is an optimal BMI for peak lipid content and barrier function.^48^

#### 2.1.3 Skin barrier

The status of the epidermal barrier depends on individual and environmental factors [e.g., epidermal hydration, transepidermal water loss (TEWL), and sebum excretion].^50^ Epidermal lipids in the interstitial fluid act as a barrier against water and electrolytes.^51^ Obese individuals show a decrease in epidermal moisture measured in the anterior forearm, with an inverse correlation between BMI and water.^52^ Moreover, the obese have larger skin folds and thicker layers of fat in other areas, which causes them to sweat more.^53^ Although the obese are more likely to be inadequately hydrated in a representative sample from the U.S.,^54^ the effect of hypohydration does not affect cutaneous vasodilation and local sweat rate any different than it does individuals with low adiposity.^55^

### 2.2 Dermis

#### 2.2.1 Thickness

Obesity changes the overall thickness of the skin and particularly the thickness of the dermal layer.

Derraik et al. and others^56^^–^^59^ demonstrate these skin thickness variations at different anatomical sights and between sex, ages, and BMI levels. Generally, skin thickness will also vary depending on anatomical region, and sometimes these parameters are combined. For example, men have a higher dermal thickness in the thigh. Several studies have shown that both epidermis and dermis thickness decrease with age.^56^^,^^57^ Tan et al.^58^ demonstrate that there is a linear increase in dermal thickness up to the age of 20 and dermal thinning afterward. There is also variation at the age where dermal thinning occurs between men and women. It is observed that in women thickness remains relatively unchanged until age 50, whereafter it starts to decline.^59^

In both men and women, the dermal layer becomes thicker with corresponding BMI.^56^ Jain et al.^35^ concluded that females have higher skin thicknesses compared with men and that in both sexes, this positive correlation allows the use of BMI to estimate tissue thickness. The only set of confounding data for the dermal layer of skin was reported by Smalls et al.,^60^ and they showed that the dermal thickness at the female shoulder is negatively correlated (r=−0.46, p=0.03) with BMI. Nevertheless, the low correlation coefficient was only significant among low population sample (n=22) subjects with a high distribution (59%) of individuals in the younger age category (21 to 30 years). Findings by Matsumoto et al.^61^ report a significant increase in dermal thickness in the abdomen, thigh, and upper arm of overweight males compared with the control group. On the other hand, Black et al.^62^ show no significant changes in skin thickness even though the girth and surface area of the forearm increase with BMI. We need to point out that these authors did not differentiate between skin layers but just pointed to other studies focusing on the dermis as a confirmation of their results.

#### 2.2.2 Vasculature

The dermal vasculature contains arterioles, venioles, and capillaries feeding the dermal tissue and living epidermal layer at the dermal/epidermal interface.^63^ Dermal vascular changes occur as skin physiology is altered through obesity. Dermal capillary density (the amount of blood in terms of the number of capillaries per area of tissue) and cutaneous blood flow (the volume of blood per unit time) are major determinants of total blood content. They vary with obesity and are consequently major contributors to the absorption of this layer. Decreased dermal capillary density with increasing obesity has been shown across different studies in different populations.^47^^,^^64^^,^^65^

#### 2.2.3 Transepidermal water loss

As opposed to sweating, which is an active thermoregulatory process to maintain homeostasis, TEWL is a passive mechanism in which water evaporates through the epidermis in an imperceptible way. Although water loss occurs through the epidermis, hence the name, it is the lower sublayers that act as the main source of water in the mass transport diffusion models used to calculate TEWL.^66^ So in terms of optical properties, the dermis and subcutis may also be affected by the migration of water, especially at the longer wavelengths of the near-infrared (NIR) range.^67^

Higher TEWL is a sign of an impaired skin barrier yielding faster rates of evaporation and lower concentrations of water withheld at the stratum corneum of the epidermis.^68^ Given that water loss measured with TEWL is diffusing out of the lower sublayers, we have assumed in our spectral model that the higher values of TEWL measured in the obese skin drive a faster depletion rate of water volume fraction in the dermis (ignoring replenishing rates). Yet to the authors’ knowledge, no direct connections between TEWL and water volume fraction of the dermis and subcutis have been reported in the literature. There are several well-established devices and techniques used to measure TEWL,^49^^,^^69^^,^^70^ yet it is important to be cognizant of the variability among measurements caused by environmental factors such as temperature, humidity, and ventilation.^71^ These factors, alongside those inherent to each subject such as genetic factors, gender, food water consumption, and health status, will impact the level of TEWL on any given day.^72^ Taking multiple time measurements of TEWL, one can integrate the volume changes with respect to time and reach a concentration of retained water.^66^ In this case, higher TEWL will yield less concentration of water retained. Estimates for average TEWL lie between 300 and 400 mL, yet there is no “normal” value established, especially since these measurements have been shown to be site-specific.^73^^,^^74^ Kottner et al.^75^ did a systematic review and reports the lowest TEWL for breast skin and the highest for the axilla. However, in the obese, Rodrigues et al.^69^ reported the largest TEWL changes were seen in the dermal skin of women’s breasts, with about a 116% monotonic increase in TEWL of the dermis in this anatomical skin region noted between the normal, overweight, obese, and severely obese subjects. Interestingly, the TEWL values in the epidermis of the face, forehead, and abdomen were lower in healthy weight compared with overweight individuals. However, it was higher in overweight individuals compared with obese and severely obese individuals with a 10% to 59% variation in the face. In addition to the difference in TEWL with the level of obesity, there are also differences in the measurement method. For example, the TEWL values of the forearm from two separate studies showed discrepancies in trends and in percent differences between obese and nonobese by the method. Specifically, Löffler et al.^70^ showed ∼67% difference in TEWL using Tewameter (TM210, Courage and Khazaka, Cologne, Germany) between obese and nonobese groups, whereas Guida et al.^76^ reported a −41.62% difference in TEWL using a VapoMeter (SL03 Delfin, Delfin Technologies Ltd., Kuopio, Finland), resulting in differences of about 42% more water between obese versus nonobese. Further, a large Brazilian cohort (n=1339) measuring values of the anterior forearm stratum corneum demonstrated an inverse relationship between BMI and hydration levels.^52^ Moreover, measurements were taken at the forearm of obese children also show ∼101.6% increase in TEWL compared with nonobese children.^77^ Although higher TEWL has been linked with impaired epidermal barrier function, Effendy et al.^70^ concluded that although TEWL was correlated with high BMI, epidermal barrier function was not affected. Instrumentation combined with the high dependency on environmental and subject factors, like skin area, show TEWL variability among values reported in the literature.^75^^,^^78^ Nevertheless, the majority of studies agree that people with higher BMI will have larger TEWL.^49^^,^^69^^,^^70^

#### 2.2.4 Collagen

Differences in collagen fiber thickness among the severely obese are measured by Sami et al., where a 23% higher thickness level is reported relative to their nonobese counterparts. This would likely result in higher scattering. Although Sami et al.^79^ reported no significant differences in collagen density, decreased collagen density across body regions was observed among overweight Japanese males,^61^ more specifically the upper arm, abdomen, and thigh with a 6.5%, 16.7%, and 10.4% lower, respectively, relative to the nonobese group.^61^ For the lower dermis of these anatomical regions, collagen density was lower even more among the obese group (18.3%, 30.2%, and 29.3% in the upper arm, abdomen, and thigh, respectively).^61^ Rapid weight loss among the obese impacts their dermis characteristics including collagen content.^80^ Light et al.^81^ reported postbariatric patients’ skin to have a loose extracellular matrix due to collagen resorption and elastin degradation. Orpheu et al.^82^ also found collagen (10% to 26%) depletion in the abdominal tissue postbariatric patients, yet no difference was seen in elastic fibers when compared with never-obese patients. These overall collagen changes due to obesity will affect the optical properties by lowering the scattering and the autofluorescence of the dermis.

#### 2.2.5 Autofluorescence

The primary contributors to autofluorescence in the dermal layer are collagen and elastin, which make up the majority of the dermis by weight.^44^^,^^83^ As obesity leads to changes in collagen in the dermis, there are autofluorescence changes as well. The most studied change is that of advanced glycation end-products (AGEs). AGEs are primarily associated with collagen and include specific molecules such as pentosidine, glucosepane, and carboxymethyl-lysine, which are almost exclusively found in the dermis.^84^ AGEs increase directly as a function of collagen turn-over and cause collagen cross-linking.^85^^,^^86^ Pentosidine-like AGEs were found to be characterized by a peak autofluorescence between 350 and 400 nm, however, Nomoto et al. demonstrated a changing pentosidine emission spectra as a function of excitation wavelength.^87^^,^^88^ This change in emission spectra as a function of excitation wavelength is also found in collagen autofluorescence. While it is well known that AGE concentration has a direct positive relationship with chronological age, BMI also has a strong increasing relation to AGE concentration and thus its autofluorescence spectra. This relationship can be explained through the association of obesity and the remodeling of collagen in the extracellular matrix, which involves AGEs.^89^ Corstjens et al.^90^ determined, using an excitation wavelength of 370 nm and measuring an emission wavelength of 440 nm, that there was a direct increasing relation between autofluorescence intensity of the ventral forearm and BMI. This pair of excitation and emission wavelengths is known to excite collagen-specific AGEs.^91^ Paolillo et al. corroborated this conclusion, as a direct increasing relationship was found between autofluorescence and BMI at other AGE specific wavelengths.^92^ This study utilized an excitation between 300 and 420 nm (peak at 350 nm) and collected data by analyzing the ratio of emitted light between 420 and 600 nm with the excitation intensity. Ahmad et al.^93^ also reported a similar increasing relationship but noted that the BMI-Skin autofluorescence relationship was only significant for men. However, if obesity status was measured as a waist to hip ratio, then a direct increasing relationship between this measure to AGE-related skin autofluorescence was shown to exist in both sexes. Additionally, interventions to reduce weight in obese adults (by energy restricted diets or bariatric surgery) reduced AGEs.^94^

### 2.3 Subcutis

#### 2.3.1 Thickness

For most people, subcutis, often termed subcutaneous adipose tissue (SAT), increases with age, although gender and ethnic differences also exist.^95^ Adult females showed a thicker SAT than males, yet both showed increasing trends with BMI.^56^ SAT is typically higher in African American youth, whereas East Asians have the lowest accumulation of SAT. Severe obesity, aging, low physical activity, and other factors impair SAT resistance to dysfunctional changes and promote the development of metabolic disorders.^96^ The obesity-related adipocyte expansion contributes to tissue oxygenation changes, albeit the literature is not definitive with conflicting results.^97^ Changes in the subcutis layer of the skin thickness have been shown to increase among the obese foot, showing a 22.6% and 14.5% relative increase in the dorsal region of the foot and under the scaphoid, respectively.^98^ Moreover, the subcutis layer appears to have the largest increase in thickness relative to BMI, with a 327% increase accounting for an increase in 20 BMI units.^56^ Among obese men, the abdomen and femoral fat cell size demonstrate a 28.9% and 14.9% relative increase, respectively.^99^ Comparing severely obese versus overweight/obese groups, there is a 27.4% increase in adipocyte size, which would also cause an increase in forward scatter.^100^

#### 2.3.2 Lipids

The optical properties of mammalian fat have been studied.^101^^,^^102^ In general, fat differs from lipids in SAT in that SAT contains about 80% fat, with the remaining 20% composed of water, protein, and minerals.^103^ Spinelli et al.^104^ analyzed the lipid content of the subcutis of the breast, finding a 0.78% increase in lipid concentration per increase in BMI unit. Specifically, comparing the lipid concentration values from nonobese versus obese (BMI: 20 versus 35) resulted in >20% relative increase. Taroni et al.^105^ calculated the lipid percent in the forearm and found that it yielded about a 34% increase in lipid concentration with obesity. Similar increases in subcutaneous lipid mass content are seen in the abdomen, although differences exist between men and women, with men having a greater relative increase in lipids with obesity of 105% versus 60%, respectively.^106^

#### 2.3.3 Water

Spinelli et al.^104^ showed an inverse relationship between water and BMI. A 1.1% decrease in water concentration per BMI unit was seen in the subcutis skin layer of the breast.^104^ Similar to epidermis and dermis, variation exists in the subcutis depending on body regions as well. The abdomen, forearm, and breast of the subcutis for a person with a high BMI compared with a person with a lower BMI showed a relative drop in water concentration of 16%, 59%, and 87%, respectively. This is somewhat consistent with Taroni et al.,^105^ who demonstrated that the dominating absorption coefficient spectral features in a forearm are due to hemoglobin and water for people that have a thinner SAT thickness, whereas lipids are the main constituent for these absorption features in the forearm of a subject with a thicker SAT such as is found in the obese. The smallest percent changes in subcutis water seen in the abdomen could be attributed to the study population demographics, which compared only overweight versus obese individuals rather than normal-weight subjects.^107^ Consistent with the above observations, obese persons also show an increase in abdominal subcutaneous water content as they lose weight.^107^ However, in diseased states such as lymphedema, skin water content should not be regarded constant, as fluid accumulation can dynamically vary depending on skin progression. For instance, edema will begin in the dermis in the early stages and travel across into the deep fascia at later stages leading to thickening of the skin and subcutis.^72^

#### 2.3.4 Autofluorescence

The autofluorescence of the subcutis as a function of BMI has not been explored in depth. This is likely due to the difficulty of visible wavelength light being able to penetrate down to and return from the subcutis. Swatland et al. found that porcine subcutaneous adipose fluorescence features an emission peak at 510 nm when excited by 365 nm light. Thus, collagen type III, which also shares this peak, is likely the significant contributor to autofluorescence at this layer.^108^ As a collagen-containing layer, it is possible that many of the relationships found between BMI and autofluorescence in the dermis hold in the subcutis. However, Odetti et al.^109^ determined that with excitation wavelengths of 335 nm (em: 385 nm) and 370 nm (em: 440 nm), there was no significant correlation between BMI and fluorescence observed. This study was conducted on patients with an average age of 61 (standard deviation: 2 years). Because of the change in the skin with age, it is not clear that the results from this higher-than-average age of participants would hold across individuals at all age ranges. From the above analysis, it is known that there are direct relationships between the formation of AGEs and chronological age as well as between collagen density and chronological age.^59^^,^^110^

### 2.4 Effects of Obesity on Skin Thickness, Blood Flow, and Chromophore Concentrations

At times, authors document skin changes (optical properties) without differentiating the layers where the information was probed. Here, we report some of the studies that present findings related to obese levels, yet whose results cannot be set to occur on one specific layer but rather across skin tissue layers.

#### 2.4.1 Total skin thickness

When comparing bulk skin thickness and the measured scattering coefficients *in vivo*, Kono and Yamada^111^ found that thinner skin had a higher scattering coefficient, both for different body parts and gender. Iacopi et al.^112^ reported an increase in thickness of skin and subcutaneous tissues at the lower limbs of obese patients.

#### 2.4.2 Vasculature and dynamic changes in the obese

In general, capillary density decreases as BMI increases and a microcirculation study by Altintas et al.^47^ showed changes to capillary recruitment and capillary network density with changes in BMI. Their findings show an inverse relationship between capillary density and BMI that agrees with previous studies.^64^ Altintas et al.^47^ noted an 18% reduction in capillary density in the forearm of overweight individuals compared with their healthy, lean, and age-and-sex-matched counterparts. The largest drop in capillary density is seen in the SAT and visceral tissues of the severely obese, where Gealekman et al.^100^ reported a 54% decrease in density relative to the overweight/obese group. Although not as large, Czernichow et al.^64^ also reported a decrease in capillary density in the middle fingers of overweight individuals. After venous congestion, the obese group only showed a 1.8% increase in density from their baseline value, whereas the nonobese group increased by 8.7%.^65^ This impairment to capillary recruitment agrees with studies done on children^113^ as well as women, in which the obese experienced a reduced percent increase in capillary density compared with nonobese counterparts.^114^ On the contrary, Czernichow et al.^64^ conducted a larger study (n=120) in which they report an increase in capillary recruitment among the insulin-sensitive overweight group.

In general, cutaneous blood flow increases as BMI increases as cutaneous blood flow has been shown to be higher in the skin of the obese.^47^^,^^49^^,^^113^ Specifically, Mori et al.^49^ reported BMI to be significantly correlated with cutaneous blood flow in American women. Further, in obese children, there appeared to be significantly elevated cutaneous baseline blood flow and peak blood flow compared with healthy control subjects.^113^ The face and forearm appeared to demonstrate similar relative percent increases in cutaneous blood flow with obesity, with each being 25.2% and 23.6%, respectively.^47^^,^^49^ Löffler et al.^70^ reported larger percent increases in forearm blood flow of the obese, namely 37.5%. Chin et al.^113^ reported a 123.4% increase in the nailfold of obese children.

In general, subcutaneous blood flow decreases as the BMI increases. Adipose tissue blood flow (ATBF) is lower in obese individuals both after a meal and during the fasting state.^115^ Further, forearm blood flow has been shown to be significantly less in the obese while performing a high-intensity exercise.^116^ Moreover, those with higher BMI have been shown to have a lower SAT blood flow.^99^^,^^117^^,^^118^ A series of studies have looked at quantifying blood inside of the subcutis layer, specifically the changes seen with different levels of obesity, which seem to yield lower blood concentrations in this tissue layer due to reduced blood flow.^99^^,^^117^^,^^118^ Specifically, while no significant differences were seen in femoral adipose tissue, ATBF was reported to be greater in the abdomen of lean versus obese men.^99^ Corroborating results reported by Larsen et al.^117^ indicate that fatty blood flow (FBF) significantly decreased as fatty tissue increased, with about 39.5% decrease in FBF. Reports from Bolinder et al.^118^ demonstrated similar findings with a 38.8% relative percent decrease in abdominal ATBF. Moreover, a reduction in blood flow was noted both while fasting and after a meal.^115^^,^^118^ Negative trends with respect to BMI were similar across age groups, although percent differences varied.^115^ A more recent study by Mitrou et al.^119^ showed a more drastic decrease in anterior abdominal wall blood flow, with a 50.9% reduction in ATBF of severely obese versus nonobese women.

#### 2.4.3 Concentration of total hemoglobin and bilirubin decreases as BMI increases

Beyond the blood volume and flow, Akter et al.^120^ found the chromophores in the blood, like total hemoglobin, significantly increase relative to normal subjects among obese men and women. The bulk skin of the female breast showed total hemoglobin decreased by 34.5% with obesity.^104^ Similar trends were seen in the face of women, where deoxy-hemoglobin content significantly decreases, yet no significant differences were seen in oxy-hemoglobin.^49^

Bilirubin in obese was shown to be 5.5% lower relative to their nonobese counterparts.^121^ Corroborating effects were reported by Andersson et al.^122^ while analyzing the effects of weight loss, where bilirubin actually increased as individuals lost weight. Beta-carotene is mainly located in the epidermis,^123^ usually with concentrations of about 0.4 nmol/g in wet tissue,^124^ with the highest levels occurring in the skin of the forehead and in the palms of the hands.^125^ However, carotenoids follow the lipid metabolic pathways and will also be found circulating in the vasculature, hence they may be also present in the subcutis.^126^ In blood, beta-carotene levels in plasma are correlated with its concentration in the skin.^127^ Moreover, researchers have demonstrated that the obese have 50% less beta-carotene in the adipocytes isolated from abdominal tissues.^128^ In obese boys, Decsi et al.^129^ show that the chromophore beta-carotene in the blood is about 51% lower when compared with the nonobese group. Although beta carotene is a strong chromophore that may be present throughout any of the skin layers, vasculature, and subcutis, there is confounding evidence as to which skin layer dominates in concentration. Thus, we have excluded this chromophore from our simple linear models that implement oxy, deoxy, water, fat, and melanin. This chromophore could nevertheless be added with an additive term containing the expected concertation and absorption coefficient in Eq. (2).

### 2.5 Construction of an Optical Propagation Skin Model

#### 2.5.1 Relative percentage change

We have revisited prior studies to understand how the anatomical and physiological changes that occur in the skin of the obese affect its optical properties. The literature values reported for the abdominal skin and vasculature among groups of low and high BMI were tabulated in Table 2 and a relative percentage change was calculated using the following equation:

$$\text{Relative percentage change}=\frac{\left(\text{Value highest BMI}-\text{Value at lowest BMI}\right)}{\text{Value at lowest BMI}}.$$ (1)

**Table 2:** Abdominal values and percent changes.

Skin layer	Parameter	Anatomical region and tissue type	Instrument/method	Population size	Value at lowest BMI	Value at highest BMI	Relative % change (%)
Epidermis	Cell size	Abdominal keratinocytes^48^	Ki-67 immunostaining and microscopy	n=50, females	4.7	11.9	153
	Water	Abdominal epidermal hydration—inverted U^69^	Moisture meter SC	n=89, females	32.1	26	−19
	Lipids	Abdominal cholesterol^48^	Cholesterol E-test	n=50, females	2.4	1.4	−41.70
		Abdominal fatty acids^48^	NEFA C-test		3.8	1.95	−48.70
Dermis	Blood	SAT and visceral tissue capillary density^100^	Immunohistochemistry (IHC) and microscopy ($\text{lumens}/{\mathrm{mm}}^{2}$)	n=17	OW/OB: 94.27	MOB: 43.3	−54
	TEWL	Abdomen^69^	Tewameter TM300	n=89	6.4	7.9	23.44
	Collagen	Abdominal (epigastrium) ^82^	Image analyzer system (Kontron Electronic 300, Zeiss, Germany)	n=80	58.6	46.4	−21
Subcutis	Blood	Abdominal subcutaneous ATBF^115^	Xe-clearance	n=24	3.4	1.7	−50
	Water	Abdomen^107^	Dielectric constant	n=27	27.8	23.3	−16.20
	Melanin	Eumelanin—abdominal visceral adipose tissue measured as PTCA in ng/μL^46^	LC-UV-MS, immunohistochemical staining, and L-[U-14C] tyrosine assay	n=10	0.05	0.19	280
	Fat (lipids)	Abdominal subcutaneous fat (Kg) ^106^	Magnetic resonance imaging	n=173, males	2.08	4.26	104.80
	Collagen	Collagen V^130^	RT-PCR and IHC	n=17	10.9	18	65.10

To summarize our work, we have provided Table 3 with the major trends found in the literature, however, the complete detailed list can be found in the Supplemental Materials. Whether it is a chromophore, a fluorophore, or a scatterer, these percentage changes directly reflect the increase/decrease of their size and content. Thus, we have used these percentage changes to alter optical properties values of abdominal skin and subcutis tissue layers from a baseline to a consequent value. The preliminary results provide insight into the absorption and reduced scattering coefficient trends as BMI is increased.

**Table 3:** Trends in optical properties affected by high BMI.

	Absorption coefficients’ contribution from major chromophores			
	Blood $\mu_{a}$	Water $\mu_{a}$	Lipid $\mu_{a}$	Melanin $\mu_{a}$
Epidermis	na	↓	↓	nc
Dermis	↓^a^	↓	nc	na
Subcutis	↓	↓	↑	↑
	Reduced scattering coefficients’ contribution from major scatterers			
	Size ${\mu_{s}}^{\prime }$	Water ${\mu_{s}}^{\prime }$	Lipid ${\mu_{s}}^{\prime }$	Collagen ${\mu_{s}}^{\prime }$
Epidermis	↑	↓	↓	na
Dermis	nc	↓	nc	↓
Subcutis	↑	↓	↑	↑

a Confounding results.

#### 2.5.2 Absorption

A generic tissue model^131^ is used to determine the effect of the changes in optical properties of the skin due to obesity. The total absorption coefficient of each skin layer can be represented from the respective contributions (volume fractions) of the major chromophores found in each tissue layer. An example of using this approach can be seen as follows:

$$\mu_{a}=BS\mu_{a.\mathrm{oxy}}+B(1-S)\mu_{a.\text{deoxy}}+W\mu_{a.\text{water}}+F\mu_{a.\text{fat}}+M\mu_{a.\text{melanosome}}.$$ (2)

**Fig. 2:**
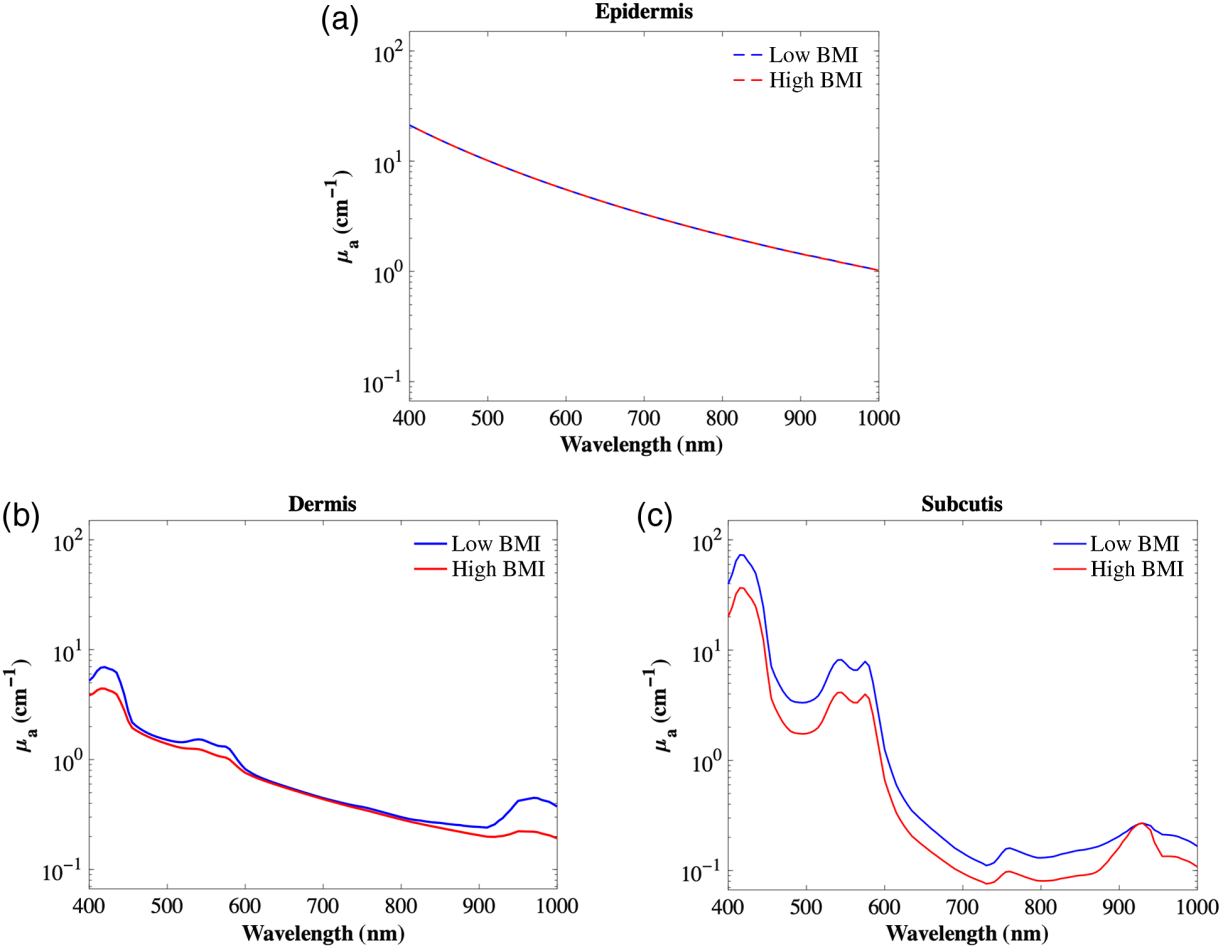
Model incorporates major abdominal chromophore’s volume fractions changes from a low BMI (baseline value) to a higher BMI (Table 4). The resulting plots model absorption coefficient for the (a) epidermis, (b) dermis, and (c) subcutis in the 400 to 1000 nm wavelength range.

We have adapted this equation to best represent the abdominal skin of low and high BMI groups. Using the values reported in the literature (summarized throughout the tables of this paper), we can calculate relative % change and directly alter the coefficients of these governing parameters. The model parameters are shown in Table 4 and the results are shown in Fig. 2.

**Table 4:** Major chromophore volume fraction changes of abdominal skin.

	Blood (B)		Hb $O_{2}$. Sat (S)		Water (W)		Lipid (F)		Melanin (M) (Fair)	
	Low BMI	High BMI	Low BMI	High BMI	Low BMI	High BMI	Low BMI	High BMI	Low BMI	High BMI
Epidermis	0	0	0	0	0.03	0.0243	0.14	0.068	0.013	0.013
Dermis	0.002	0.0092	0.40	0.40	0.65	0.1524	0	0	0	0
Subcutis	0.03	0.015	0.60	0.60	0.15	0.1257	0.8	1.64	0	0.001

#### 2.5.3 Scattering

Scattering distributes light intensity as it travels through tissue. The diffusion of photons in a random walk can be described using the reduced scattering coefficient ($\mu_{s^{\prime }}$).^37^ For some wavelength ranges of visible light and NIR (∼600 to 1100 nm), $\mu_{s^{\prime }}$ cannot be neglected, as it dominates over the absorption coefficient ($\mu_{a}$).^105^ The spectral shape of $\mu_{s^{\prime }}$ is well-established so that $\mu_{s^{\prime }}$ monotonically decreases with wavelength^131^

$$\mu_{s}^{\prime }(\lambda )=\alpha x^{-\beta }.$$ (3)

**Fig. 3:**
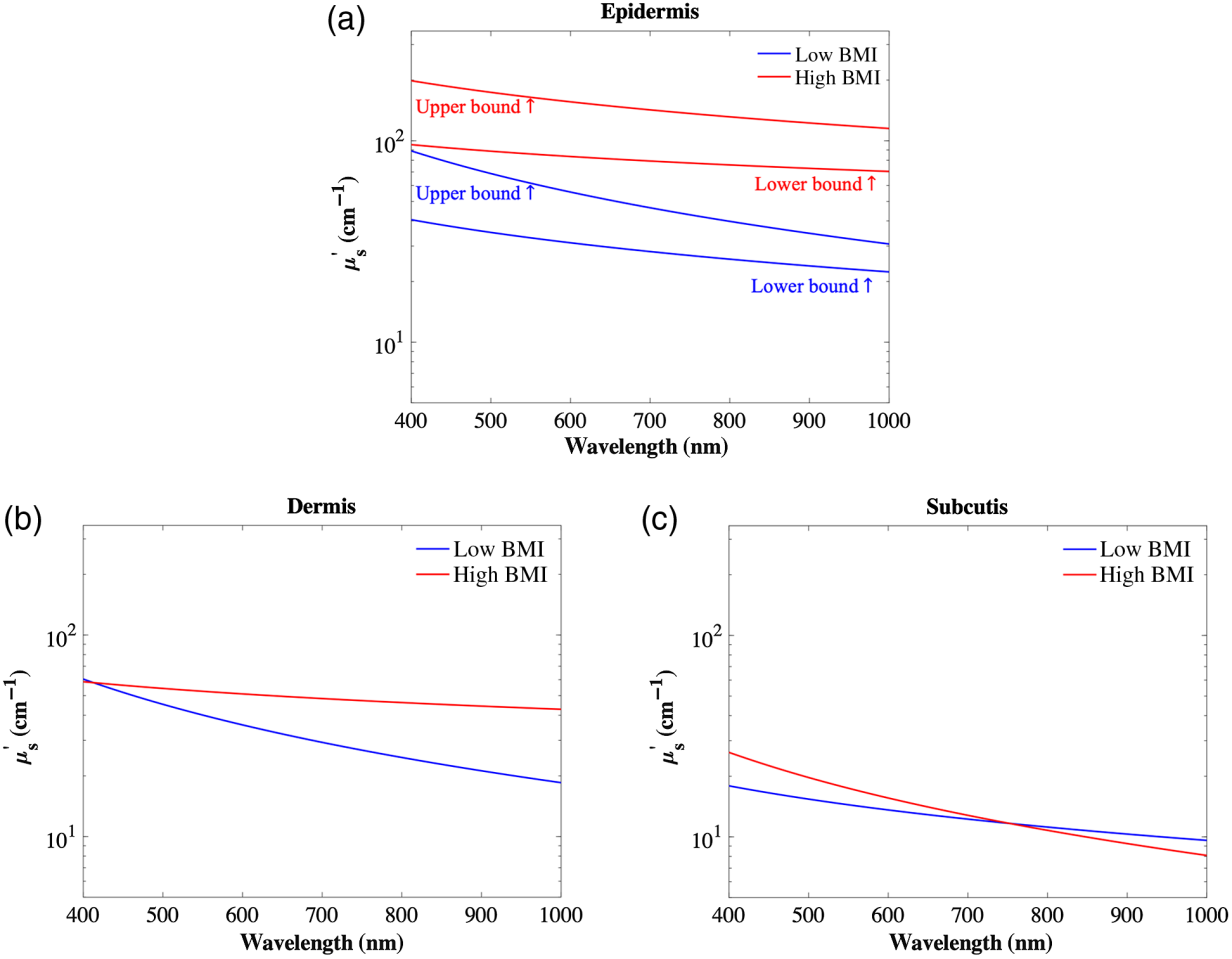
Model incorporates the abdominal reduced scattering coefficient’s fitting coefficients in skin layers (Table 5): (a) epidermis, (b) dermis, and (c) subcutis. The values are plotted for the visible and NIR wavelengths.

The fitting parameters, α, and power constant, β, are related to an average size of the scatterers’ Mie-equivalent radius and to their concentrations, respectively.^105^ We select baseline values for the fitting parameters based on those reported by Jacques et al.^131^ review of optical properties of biological tissues. Specifically, the epidermal and dermal values are adopted from Salomatina et al.,^132^ and the subcutis values are taken from Bashkatov et al.^37^ We use these to generate the low BMI spectra across skin layers (Fig. 3). Moreover, due to the large variability of epidermal scattering values that exist in the literature, we added an upper and lower bound by adopting values from Marchesini et al.^133^ Once again, using the calculated major scatterers’ relative % changes between low and high BMI groups, we alter the linear and nonlinear coefficient of Eq. (3) to create a model that estimates the reduced scattering coefficient of abdominal tissues. The model parameters are shown in Table 5 and the results are shown in Fig. 3.

**Table 5:** Abdominal skin fitting parameters (major scattering molecules).

	α (Scatterer radial size)		β (Collagen, water, and lipid)	
	Low BMI	High BMI	Low BMI	High BMI
Epidermis	68.7	174	1.16	0.596
Dermis	45.3	54.3	1.29	0.344
Subcutis	15.4	19.7	0.680	1.28

## 3 Results

The relative changes in skin anatomical and physiological components due to BMI affecting the optical properties are reported in Table 2 for all skin layers. The studies are grouped by the skin parameter that was measured and compared between low and high BMI groups. Low and high values reported throughout studies were used to calculate relative percentage changes (right-most column).

The resulting absorption coefficient spectra between 400 and 1000 nm are displayed in Fig. 2. In the epidermal layer, higher BMI does not affect the absorption coefficient when compared with lower BMI. Although invisible graphically, the highest change of 0.2% between low and high BMI occurs around 930 nm where lipids naturally peak in the spectrum. Although higher BMI exhibits a considerable reduction of lipids in this layer, fatty tissue natively expresses very little absorbance in this optical wavelength range and the overall thickness of the epidermis for both normal and obese is quite small compared with the other skin layers. The largest differences in absorption coefficient for the dermal layer are seen at the short and long wavelength extremes of the spectrum. Here, dermal changes differ up to 51% at longer wavelengths (i.e., 975 nm) and by 50% near the shorter wavelengths (418 nm) in the dermis due to the loss in absorption related to losses of hemoglobin and water concentrations at these wavelengths for the high BMI tissue. For the subcutis, there is a reduction with high BMI at nearly every wavelength for the absorption coefficient due primarily to the reduction of water content and blood flow (thus hemoglobin content). However, near a wavelength of 950 nm, they are roughly equal due to the absorption peak of lipids with both low and high BMI at this wavelength.

We used fitting equations previously presented in a review by Jacques et al.^131^ to represent the amount of scattering occurring in the abdominal tissue layers. The changes to the coefficients α and β from Eq. (3) are based on the changes in cell size, lipid, water, and collagen concentration found in the literature. The resulting reduced scattering coefficient of the low versus high BMI abdominal skin tissues are shown in Fig. 3. Across the skin layers, we used Eq. (3), which only considers Mie scattering. For all three layers, the largest changes due to high BMI occur at the extremities of the spectrum: with 274%, 131%, and 46% occurring at the epidermis, dermis, and subcutis layers, respectively. The overall reduced scattering coefficient spectrum increases in the epidermis layer primarily due to the increase in keratinocyte cell size. Although the reduced scattering is large at the shorter wavelengths of the visible spectrum, the values that we report yield a transport mean free path of ∼50  μm, which is about half of an average epidermal layer thickness, and thus the results presented here are still useful to those using the diffusion approximation of photon transport equation and its limitations. In the dermis, the reduced scattering coefficient is higher for all values within the visible spectrum mainly due to increases in fiber bundle thickness, with a negligible pivotal intersection of the two curves at shorter wavelengths around and below 400 nm. The slope in the dermis is flatter due to a reduction of the concentration of scattering particles. The overall trend for the high BMI subcutis shows higher reduced scattering in shorter wavelengths and a crossover at around 740-nm wavelength to lower values than the low BMI group because of increases in adipocyte cell size and lipid concentration. For all layers we see a general reduction in the scatter with wavelength regardless of BMI level, which is characteristic of tissue scattering because scattering particles are comparable to the size of the wavelength in the visible spectrum and due to the inverse relationship that they share.

## 4 Discussion

Epidermal absorption is governed by melanin. None of the data reviewed addresses melanin production in the epidermis to be dependent or correlated with increasing BMI. However, the high prevalence of obesity among African Americans and Hispanics makes melanin a noteworthy chromophore to adequately include in light transport models. Water losses in the epidermis were significant among the obese and agree with values from the stratum corneum of the epidermis.^52^^,^^69^ Our modeled epidermal absorption coefficient for the obese, where the volume fraction of water drops from 0.03 to 0.0243, is based on the −19% relative percentage reduction of epidermal hydration measurements conducted by Rodrigues et al.^69^ on 89 females using a moisture meter on the abdomen. It should be noted that the study reports an inverted U-shape relationship between water losses, meaning that there is a BMI level in which the water content is optimum for skin function. A similar inverted U-shape trend was seen of the epidermal lipid content of Japanese women. Horie et al.^48^ showed that as obesity increases in overweight and obese women, skin lipids (cholesterol and fatty acids) decrease, although they increase in low-weight women, yielding an optimal BMI range of around $22\;\mathrm{kg}/m^{2}$. Using their reported lipid changes, we were able to use our calculated relative percentage change of 48.7% to modify the epidermal volume fractions of lipid from 0.14 to 0.068, thus affecting absorption and scattering. In the dermis, the volume fraction of dermal water in our model reduced from 0.65 to 0.1524 after a 23.44% increase in TEWL.^69^ Among other dermal absorbers, our model’s blood volume fraction was reduced from 0.002 to 0.0092 after using the calculated relative 54% decrease in capillary density reported by Gealekman et al.^100^ The decline in blood content agrees with the majority of studies.^47^^,^^64^^,^^65^ In the subcutis, lipid content is highest compared with all other layers and increases with obesity. Our model shows a volume fraction increase (∼105%) from 0.8 to 1.64 using results reported by Janssen et al.^106^ This increase in lipids, although varying between sex and anatomical region, is prevalent across studies.^104^^,^^105^ Subcutis water, on the other hand, decreases as BMI increases. Our model’s volume fraction is reduced by 16% from 0.15 to 0.1257. One rare finding was that of eumelanin near adipocytes of severely obese individuals. While eumelanin is not usually considered to be part of the subcutis layer, authors reported a 74% increase in the concentration of pyrrole-2,3,5-tricarboxylic acid (PTCA), a melanin byproduct, between nonobese and severely obese individuals.^46^ This “appearance” of melanin is represented in our model by incorporating a volume fraction of 0.001 for the obese. Unlike the dermal layer, the subcutis exhibits a larger concentration of collagen V among the obese, especially near large blood vessels.^130^ The increase of collagen in the subcutis could lead to larger autofluorescence, although no significant correlations were linked to BMI, and thus this phenomenon is not represented in our model.

Epidermal scattering in our model increases due to the combined changes of cell size and scattering concentration, which increase the α coefficient while decreasing the β, respectively. These changes in size are based on Altintas et al.^47^ who showed an increase in epidermal cell size of 6.3% in the overweight group, which would produce more forward scattering. Moreover, in the obese epidermis, an increase in skin roughness has also been associated with increased scattering and the amount of reflection in incident light.^49^ In the dermis model, α increases by 20% due to the net findings of fiber bundle thickness measurements of the papillary and reticular dermis by Sami et al.^79^ Our model’s decrease of the β coefficient for the obese dermis is attributed to losses of scatterer concentration near the abdomen, particularly those of water and collagen. The percentage losses affecting these coefficients were based on the reported changes of 23.44% and 21% showing dermal water and collagen depletion.^69^^,^^82^ Thus, scattering results are higher in the obese versus nonobese due to changes in water concentration and changes in skin collagen size, organization, and density in the skin of the obese.^61^^,^^79^^,^^81^^,^^82^

The α coefficient in the subcutis was increased by 28% in our model based on the findings of Jansson et al. and Gealekman et al., where 28.9% increase in fat cells and 27.4% in adipocytes size was noted, respectively.^99^^,^^100^ These increments agree with fat cell size increments of 14.9% extracted from the femur.^99^ While water decreases in the subcutis,^107^ it is the vast increase in lipids combined with the minor contribution of collagen V,^130^ which nearly double the value of the β coefficient from 0.68 baseline values to 1.28.

For the most part, studies show that obesity increases the thickness of many skin layers. For optical devices, this increase in thickness yields an increased optical path length. This information is important in terms of the propagation of the light photons through the tissue as they encounter more possibilities for scatter, absorption, and autofluorescence within thicker tissue, which can be predicted using Monte Carlo models of light transport that can ultimately be used for optical device design. Based on values extracted from the literature, the epidermal path length will increase in our model by 33.3% as seen in the abdomen of Japanese women,^48^ which agrees with increments of 24.5% seen in at the volar forearm.^47^ Similarly, dermal thickness is shown to increase with BMI, specifically showing an increase of 20 BMI units for a 36% increase in dermal thickness.^56^ The optical path length of our model will respectively increase in the dermis and subcutis by 35% and 227% based on the findings by Derraik et al.^56^ These increments are in accordance with other body regions where skin thickness also increases, such as those in people with diabetes that are obese. Depending on the anatomical region, optical path length changes due to increases in skin thickness (epidermis + dermis) will increase by 14.3%, 17.7%, 4.8%, or 17.4% for the rear upper arm, anterior upper thigh, anterior abdomen, or the upper outer quadrant of buttocks, respectively.^98^

## 5 Conclusion

We have completed a literature review of skin parameters that affect optical property changes across layers for individuals of elevated BMI. We grouped the parameters and sorted them according to anatomical location and instrumentation used. Moreover, we extracted parametric values for low and high BMI groups and used their relative percentage differences to create models of spectral properties (absorption and reduced scattering) for these two groups. Our results show layer-depended differences of optical properties between low and high BMI individuals. In the VIS and NIR regions of the spectrum, layer-specific absorption coefficients range between (0.08 to $37.36\;{\mathrm{cm}}^{-1}$) for obese and (0.11 to $74.5\;{\mathrm{cm}}^{-1}$) for nonobese and the reduced scattering coefficient varied between (8 to $199\;{\mathrm{cm}}^{-1}$) for obese and (8.6 to $89\;{\mathrm{cm}}^{-1}$) for nonobese. It is clear from the above discussions and the summaries depicted in the tables and figures that the absorption, scattering, autofluorescence, and optical propagation of the light through the skin change not only with obesity but with both the level of obesity (e.g., normal to overweight to severely obese) and the skin location on the body (e.g., abdomen, breast, face, forehead, foot, and hand, etc.). Future work will focus on experimentally validating these findings. Given this validation, future models and optical methods may be confidently tailored to the obese population. We expect that this, in turn, will increase the accuracy of chromophore concentration extraction and thus health-dependent biomarkers.
