# Supplementary material for: Skin optical properties in the obese and their relation to body mass index: a review
Source: J Biomed Opt. 2022 Mar 29;27(3):030902. doi: 10.1117/1.JBO.27.3.030902 (PMC8963797; doi:10.1117/1.JBO.27.3.030902)
Supplement: Supplementary file 1 [file JBO_027_030902_SD001.pdf]

Skin Layer	Parameter	Anatomical Region and Tissue Type (reference)	Instrument/ Method	Population Size	Value at Lowest BMI	Value at Highest BMI	Relative % Change
		Abdominal keratinocytes (Horie, 2018)	Ki-67 immunostaining and microscopy	n = 50, females	4.7	11.9	153.20%
		Forearm epidermal cell size (Altintas, 2016)	Reflectance-mode confocal microscopy (RCM)	n = 20	772.6	821.3	6.30%
		Forearm epidermal thickness (Altintas, 2016)	Reflectance-mode confocal microscopy (RCM)	n = 20	44	54.8	24.50%
	Size and Thickness	Abdominal epidermal area (Horie, 2018)	Hematoxylin/eosin staining, and microscopy	n = 50, females	0.045	0.06	33.30%
		Face surface roughness (Mori, 2017)	Polarized images Visioscan VC98	n = 93	1.08	1.83	69.40%
		Forearm stratum corneum moisture (de Farias Pires, 2016)	Corneometer CM820	n = 492, males	23.7	13.65	-73.60%
				n = 847, females	32.85	23.7	-38.60%
		Abdominal Epidermal Hydration – inverted U (Monteiro Rodrigues, 2017)	Moisture Meter SC	n = 89, females	32.1	26	-19%
		Breast (Monteiro Rodrigues, 2017)			45.7	69.7	52.50%
		Forehead (Monteiro Rodrigues, 2017)			49.9	46.1	-7.60%
	Water	Zygomatic area (Monteiro Rodrigues, 2017)			48.5	44.9	-7.40%
Epidermis	Lipid	Abdominal Cholesterol (Horie, 2018)	Cholesterol E-Test, NEFA C-Test	n = 50, females	2.4	1.4	-41.70%
		Abdominal Fatty acids (Horie, 2018)			3.8	1.95	-48.70%
		Abdomen - dermal thickness (Matsumoto, 2014)	DermaScanC ultrasound	n = 90, males	1.53	1.72	12.42%
		Thigh - dermal thickness (Matsumoto, 2014)			1.39	1.65	18.71%
		Upper arm - dermal thickness (Matsumoto, 2014)			1.51	1.8	19.21%
		Abdominal and thigh dermal thickness (Derraik, 2014)	Phillips IU-22 ultrasound machine	n = 140	1.7	2.3	35%
		Rear upper arm epidermis & dermis (Gibney, 2010)	Cortex DermaScanC	n = 388	2.1	2.4	14.30%
		Anterior upper thigh epidermis & dermis (Gibney, 2010)			1.7	2	17.60%
		Anterior abdominal epidermis & dermis (Gibney, 2010)			2.1	2.2	4.80%
		Outer buttocks epidermis & dermis (Gibney, 2010)			2.3	2.7	17.40%
		Dorsal foot (heel) Skin thickness [mm] (skin & subcutaneous tissues) (Iacopi, 2020)	Logiq 7pro ultrasound system	n = 120	1.11	1.4	26%
	Size and Thickness	Dorsal foot (under scaphoid) Skin thickness [mm] (skin & subcutaneous tissues) (Iacopi, 2020)			0.57	0.65	14%
		Upper back (shoulders) dermal thickness (Smalls, 2006)	DermaScanC ultrasound	n = 22	nr	nr	r = -0.46
		Abdomen (Monteiro Rodrigues, 2017)	Tewameter TM300	n = 89	6.4	7.9	23.44%
		Breast (Monteiro Rodrigues, 2017)			7.3	15.8	116.44%
		Forehead (Monteiro Rodrigues, 2017)			9.6	15.3	59.38%
		Zygomatic area (Monteiro Rodrigues, 2017)			11.7	16.3	39.32%
		Face (right cheek) (Mori, 2017)	Tewameter MPA 580	n = 93	17.6	19.4	10.23%
	TEWL	Forearm (Guida, 2010)	VapoMeter SL03 Delfin	n = 80	18.5	10.8	-41.62%
		Forearm (Loffler, 2002)	Tewameter TM210	n = 63	6.9	11.5	66.67%
		Forearm collagen content (Black, 1971)	5 mm punch biopsies	n = 16	nr	nr	no change in content, thus density must decrease
		Abdominal collagen bundle thickness (Light, 2010)	Histological staining and light microscopy	n = 10	nr	nr	decreased thickness – degraded and disorganized matrix
		Back collagen bundle thickness (Light, 2010)			nr	nr	
		Papillary dermis collagen fiber thickness [µm](Sami, 2015)	Hematoxylin Eosin, and Mallory staining and Image Analyzer	n = 30	1.78	2.19	23%
		Reticular dermis collagen fiber thickness [µm](Sami, 2015)	System (Leica Q 500 MC program)		9.32	9.03	-3.10%
		Abdominal (epigastrium) collagen concentration (Orpheu, 2010)	Image Analyzer System (Kontron Electronic 300, Zeiss, Germany)	n = 80	58.6	46.4	-20.80%
		Abdominal (hypogastrium) collagen concentration (Orpheu, 2010)			52.4	47.6	-10%
		Upper arm – upper dermis collagen density (Matsumoto, 2014)	DermaScanC ultrasound	n = 80, males	12	11.2	-6.70%
		Upper arm – lower dermis collagen density (Matsumoto, 2014)			6.4	5.2	-18.80%
		Abdomen – upper dermis collagen density (Matsumoto, 2014)			11.7	9.8	-16.20%
		Abdomen – lower dermis collagen density (Matsumoto, 2014)			7.7	5.4	-29.90%
		Thigh – upper dermis collagen density (Matsumoto, 2014)			18.3	16.4	-10.40%
		Thigh – lower dermis collagen density (Matsumoto, 2014)			12.1	8.6	-28.90%
		Thigh collagen density (Ibuki, 2018)	DermaScanC ultrasound	n = 69, males	nr	nr	low collagen density associated with high oxidative stress
Dermis	Collagen	Papillary dermis collagen density [µm] (Sami, 2015)	Hematoxylin Eosin, and Mallory staining and Image Analyzer	n = 30	53.47	56.41	5.50%
		Reticular dermis collagen density [µm] (Sami, 2015)	System (Leica Q 500 MC program)		67.12	68.72	2.40%
		Abdominal fat cell size (Jansson, 1992)	Sectioned sliced and treated with collagenase	n = 16	83	107	28.90%
		Femoral fat cell size (Jansson, 1992)			94	108	14.90%
		Subcutaneous adipose tissue adipocyte cell size [µm2] (Gealekman, 2011)	Immunohistochemistry	n = 17	1427	1818	27.40%
		Abdominal and thigh subcutis thickness [mm] (Derraik, 2014)	Phillips IU-22 ultrasound machine	n = 140	8.8	28.8	227%
		Rear upper arm SAT (Gibney, 2010)	GE LOGIQ e ultrasound	n = 388	7.7	13.5	75.30%
	Size and Thickness	Anterior upper thigh SAT (Gibney, 2010)			7.8	12.6	61.50%
		Anterior abdomen SAT (Gibney, 2010)			9.5	17.3	82.10%
		Outer buttocks SAT (Gibney, 2010)			13.7	16.6	21.20%
		Forearm lipid concentration (Taroni, 2003)	Time-resolved reflectance and transmittance spectroscopy	n = 2	64	86	34.30%
		Breast lipid content (Spinelli, 2004)	Multiwavelength time-resolved optical mammography	n = 113	54.4	66.1	21.50%
	Lipid	Abdominal subcutaneous fat [Kg] (Janssen, 2002)	Magnetic resonance imaging	n = 173, males	2.08	4.26	104.80%
				n = 101, females	2.66	4.25	59.80%
		Forearm water concentration (Taroni, 2003)	Time-resolved reflectance and transmittance spectroscopy	n = 2	32	13	-59.40%
		Breast water content (Spinelli, 2004)	Multiwavelength time-resolved optical mammography	n =113	18.9	2.4	-87.30%
	Water	Abdomen (Laaksonen, 2003)	Dielectric constant	n = 27	27.8	23.3	-16.20%
		Collagen V (Spencer, 2011)	RT-PCR and immunohistochemistry (IHC)	n = 17	10.9	18	65.10%
		Eumelanin – abdominal visceral adipose tissue measured as PTCA in ng/µl (Randhawa, 2009)	LC-UV-MS, immunohistochemical staining, and L-[U-14C] tyrosine assay	n = 10	0.05	0.19	280%
Subcutis	Other	Subcutaneous connective tissue from lower dermis – fluorescence (Odetti, 1992)	L55B Perkin-Elmer spectrophotofluorometer	n = 26	nr	nr	No significant correlation with BMI
	Size	Carotid artery vessel thickness [mm] (Vinet, 2010)	High-resolution vascular ultrasonography	n = 16	0.5	0.62	24%
		Forearm (volar) capillary density (Altintas, 2016)	Confocal microscopy gallium-arsenide laser	n = 20	6.02	4.91	-18.40%
		Finger, dorsum, capillary density (Czernichow, 2010)	Capillaroscopy -- video microscopy	n = 250	91.8	88.6	-3.50%
		Finger, dorsum capillary density (Debbabi, 2006)	Intravital video-microscopy	n = 170	OW(NHT): 68.2 ± 1.5	OW(HT) untreated 59.6	-12%
		SAT & visceral tissue capillary density (Gealekman, 2011)	Immunohistochemistry & microscopy (lumens/mm2)	n = 17	OW/OB: 94.27	MOB: 43.3	-54%
		Finger, dorsum, capillary density a rest (Francischetti, 2011)	Video microscopy	n = 125	135.2	132.3	-2.10%
		Finger, dorsum, capillary recruitment (Francischetti, 2011)			8.7	1.8	-79.30%
		Nailfold capillary recruitment (De Jongh, 2004)	Capillaroscopy	n = 28	37.4	56.2	-33.50%
	Capillary	Finger, dorsum, capillary recruitment (Czernichow, 2010)	Capillaroscopy -- video microscopy	n = 250	5.4	9.5	75.93%
		Finger Nailfold cutaneous blood flow (Chin, 1999)	Laser doppler flowmetry and capillaroscopy	n = 34, children	71.8	123.4	123.40%
		Face (right cheek) cutaneous blood flow (Mori, 2017)	laser speckle blood flow system, PeriCam PSI NR	n = 93, females	117.8	147.5	25.20%
		Forearm (volar) dermal blood cell blood flow (Altintas, 2016)	Confocal microscopy gallium-arsenide laser	n = 20	51.1	63.1	23.50%
		Forearm blood flow (Loffler, 2002)	Laser Doppler (PF 5010, Perimed)	n = 62	5.6	7.7	37.50%
		Brachial artery peak blood flow (Vinet, 2010)	Ultrasonography with Doppler	n = 16	398	229	-73.80%
		Leg blood flow (Vinet, 2010)			36.9	21.5	-14.60%
		Abdomen, gluteal region, front of the thigh fatty blood flow (Larsen, 1996)	Xe-clearance	n = 69	nr	nr	-39.50%
		Abdominal ATBF (Jansson, 1992)	Xe-clearance	n = 16	3.2	1.6	-50%
		Femoral ATBF (Jansson, 1992)			2.7	2.4	-11.10%
		Abdominal subcutaneous ATBF (Bolinder, 2000)	Xe-clearance	n = 26	1.895	1.158	-39%
		Abdominal subcutaneous ATBF (Jansson, 1998)	Xe-clearance	n = 24	3.4	1.7	-50%
		Abdominal SAT (Jansson, 1998)	Xe-clearance	n = 31	OB: 2.4	OB(T2D): 1.7	-29.20%
	Blood Flow	Abdominal wall (Mitrou, 2010)	Xe-clearance	n = 37	1319	648	-50.90%
		Forearm (Monteiro Rodrigues, 2017)	Venous occlusion Plethysmography	n = 10	14.6	10.7	-24.40%
		Forearm total hemoglobin concentration [µM] (Taroni, 2003)	Time-resolved reflectance and transmittance spectroscopy	n = 2	169	32	-81%
		Breast total hemoglobin [µM] (Spinelli, 2004)	Multiwavelength time-resolved optical mammography	n = 113	17.4	11.4	-34.50%
		Face (right cheek) [cell/mm3] (Mori, 2017)	NIRS- based tissue-blood oxygenation monitor, BOM-L1TRSF	n = 93, females	7.3	7.33	-4.30%
		Circulatory hemoglobin [mg/dL] – inverted ushaped (Ghadiri-Anari, 2014)	Fasting blood samples	n = 406	13.2	13.5	2.3% non.sig. increase
		Blood hemoglobin concentration [g/dL] (Hung, 2019)	Blood test - Hb positive correlation in pre- and post-bariatric surgey	n = 221	13.2	14.2	7.60%
		Circulatory hemoglobin (Akter, 2017)	Cyanmethemoglobin Method	n = 200	nf	nf	significantly increased
Vasculature	Hemoglobin	Glycosylated hemoglobin (A1c, or HbA1c) (Das, 2014)	Boronate affinity method	n = 120	nf	nf	positive correlation with BMI
		Glycosylated hemoglobin (A1c, or HbA1c) (Das, 2016)	Boronate affinity method	n = 180	(r=0.255, 0.37 and 0.39 in control, overweight and obese respectively)		positive correlation with BMI. increasing r with obesity group
